# Non surgical management for massive gastric lipomatosis

**DOI:** 10.1016/j.ijscr.2019.11.047

**Published:** 2019-12-03

**Authors:** Jaime Solano, Gabriel Herrera, Manuel Cadena, Luis Felipe Cabrera, Efrain Isaac, Mauricio Pedraza

**Affiliations:** a Department of General Surgery, Fundación Santa Fe de Bogotá, Bogota Dc, Colombia; b Department of Gastroenterology and Endoscopy, Fundación Santa Fe de Bogotá, Bogota Dc, Colombia; c Deparmten of Ocologic Surgery, Fundación Santa Fe de Bogotá, Bogota Dc, Colombia; d Departmen of General Surgery, Universidad El Bosque, Bogota, Colombia

**Keywords:** Gastric, Lipomatosis, Lipoma, Gastrectomy and subepitelial

## Abstract

**Background:**

Gastric lipomatosis (GL) is defined as a lipomatous lesion with diffuse infiltration of the submucosal layer by adipose tissue with multiple lesions and must be differentiated from gastrointestinal lipomas, which are solitary submucosal masses composed of well-differentiated adipose tissue surrounded by a fibrous capsule. GL are uncommon, represents 5 % of gastrointestinal tract lipomas and less than 1∼3 % of all gastric tumors.

**Case presentation:**

A 65-year-old woman, who underwent non-operatively approach by Gastric lipomatosis. Non-specific gastrointestinal symptoms and endoscopic examination ruled out the possibility of mucosal ulceration or bleeding. Endoscopic ultrasonography and abdominal CT that confirmed gastric lipomatosis.

**Discussion:**

Gastric lipomas are usually small, solitary and asymptomatic, and are hence often detected incidentally, surgical management in asymptomatic patients still be a in debate.

**Conclusion:**

Non operative management in massive gastic lipomatosis is appropiate when the patient is asymptomatic without mucosal ulceration, regardless of the amount of gastric lipomas. Avoiding patient the morbidity of a total gastrectomy.

## Highlights

• Massive gastric lipomatosis represent 5% of gastrointestinal tract lipomas and less than 1% of all gastric tumors.
• Early diagnose and multidisciplinary follow up, is reflected is an adequate evolution.
• Asymptomatic massive gastric lipomatosis, could be follow with endoscopy.

## 1 Introduction

Gastric lipomatosis(GL) is defined as a lipomatous lesion with diffuse infiltration of the submucosal layer by adipose tissue with multiple lesions and must be differentiated from gastrointestinal lipomas, which are solitary submucosal masses composed of well-differentiated adipose tissue surrounded by a fibrous capsule. Gastric lipomas are uncommon, and account for only 5 % of gastrointestinal tract lipomas and less than 1∼3 % of all gastric tumors. However, gastric lipomatosis is a rare condition, with only ten previously reported cases in the scientific literature. The present report describe the case of gastric lipomatosis managed with non surgical approach with optimal clinical results [[1], [2], [3], [4]]. This work has been reported in line with the SCARE criteria [21].

## 2 Case report

**Fig. 1:**
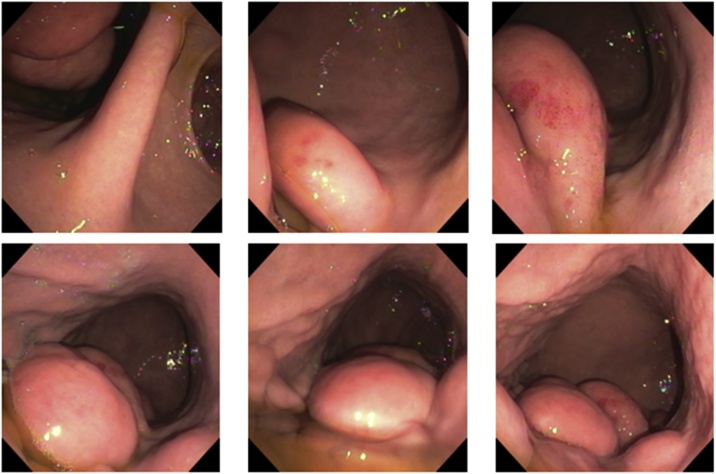
Digestive endoscopy: º masses around the gastric wall.

**Fig. 2:**
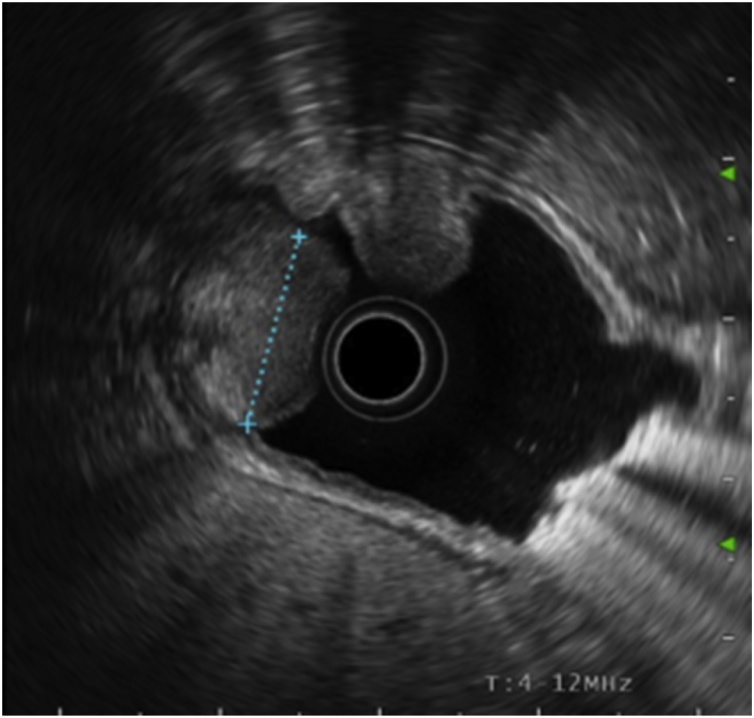
Endoscopy ultrasound: huge fat-containing mass lesion well-defined, homogeneously hyperechoic with a maximun size of 27 × 17 mm.

**Fig. 3:**
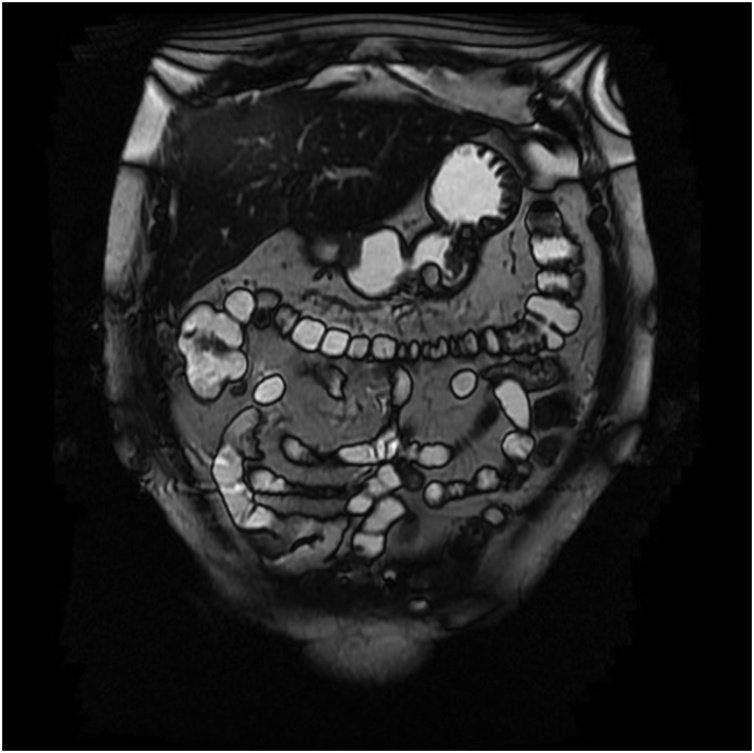
Magnetic resonance enterography: multiple fat-containing masses around the gastric wall.

A 65-year-old woman was admitted with intermittent epigastric pain for the previous 24 months. She had no significant past medical or family history. Upper endoscopy revealed multiple subepitelial masses around all the gastric wall without ulcerations of active bleeding (Fig. 1). Laboratory tests where unremarkable. The patient had no personal history of gastrointestinal bleeding. A physical examination of the abdomen did not reveal any masses. Endoscopic ultrasonography (EUS) demonstrated that the lesions originated in the submucosal layer, were all well-defined and were homogeneously hyperechoic with a maximun size of 27 × 17 mm (Fig. 2). An MRI enterography showed multiple fat-containing masses around the gastric wall. The lesions appeared as well-circumscribed areas of fat density with an attenuation ranging from -70 to -120 Hounsfield units (Fig. 3). There was no evidence of lymph node enlargement. Endoscopic biopsy with a polipectomy loop showed a benign mesenchymal tumor lesion of submucosal location, constituted by a proliferation of mature adipocytes, with some fibrous septa without cellular atypia, necrosis or mitosis. The patient was managed non-operatively approach with no development of bleeding at follow up.

## 3 Discussion

Gastric lipomas are rare, bening tumors that on edoscopic examination present a smooth, well defined margin, often present as an oval or spherical submucosal mass composed of well differentiated adipose tissue surrounded by a fibrous capsule [[3], [4], [5], [6]]. Gastric lipomatosis is characterized by multiple gastric lipomas or diffuse gastric infiltration on submucosal or subserosal layer by adipose tissue [[7], [8], [9]]. Previous reports described diffuse infiltration of the submucosal layer by adipose tissue as a pathologic process and suggested that the term “lipomatosis or lipohyperplasia of intestine” should be replace the term “lipoma of intestine” [1,2,10,11].

The etiology of gastric lipomatosis remains to be established. Postulated etiological factors include embryonic displacement of adipose tissue, congenital predisposition, degenerative disease with disturbance of fat metabolism, post-chemotherapeutic fat deposition, chronic irritation such as chronic inflammatory bowel disease, low-grade infection and hamartomatous syndromes [[12], [13], [14]]. Cabaud and Harris [15] proposed that a fatty lesion with an annular nature and complete lack of encapsulation is a fatty infiltration rather than a true neoplasm. They described a pathological process whereby mechanical propulsion of the mucosa and submucosa downstream into the lumen of the bowel might increase the submucosal area which is then filled by fat. No report has described malignant transformation of gastric lipomas [1,2,[11], [12], [13]].

Gastric lipomas are usually small, solitary and asymptomatic, and are hence often detected incidentally. Hundreds of gastric lipoma cases have been reported in the medical literature. Although lipomas are occasionally detected within the gastrointestinal tract and account for 4∼8 % of all gastrointestinal tract tumors, gastric lipomas are uncommon. According to a survey of gastric submucosal tumors that were surgically resected during a 2 year period in Korea, gastric lipomas accounted for 0.9 % of all gastric submucosal tumors. Approximately 75 % of gastric lipomas are located in the antrum. In addition, approximately 90∼95 % of gastric lipomas are located in the submucosal layer, with the remainder in the subserosal layer [[1], [2], [3],[18], [19], [20]].

Clinical symptoms are related to tumor size and location, and the development of complications. The most common clinical presentation (50 %) is reported to be upper gastrointestinal bleeding caused due to ulceration of the tumor [[1], [2], [3]]. Obstructive symptoms including vomiting and gastrointestinal obstruction are also frequent (42 %) [1,15,16,19]. The development of complications including gastrointestinal bleeding caused by ulceration and gastrointestinal obstruction can be manifested clinically when gastric lipomas are larger than 2 cm [1,12].

The medical literature contains few reports of gastric lipomatosis [1,2]. We found 10 case reports describing multiple gastric lipomas, and 1 case report describing diffuse depositions of adipose tissue that were not encapsulated. Eight of those 10 patients first presented due to upper gastrointestinal bleeding, and the other 2 patients had non-specific gastrointestinal symptoms similar to this case. Although gastrointestinal bleeding generally presented as chronic blood loss, 2 patients had acute severe blood loss resulting in emergency presentations. Two of the 10 patients died due to massive gastrointestinal bleeding. The 9 case reports of multiple lipomas described encapsulated tumors. 1 case describe a patient with lipomatosis that showed extensive gastric involvement and required surgical removal because of symptoms, contrary to our case, with extensive gastric lipomatosis but in an asymptomatic patient. Although a previous report also described a case featuring extensive gastro-duodenal involvement, that patient was asymptomatic [1,2,8,9,11,17]. That patient was followed-up for 5 years and showed good recovery and no disease progression.

Abdominal CT is the imaging method of choice to determine the specific nature of a lipoma, the extent of the disease and for follow-up [[1], [2], [3],^9^,^12^]. A homogeneous gastric submucosal tumor with an attenuation of between −70 and −120 Hounsfield units has been reported as a definitive finding for the diagnosis of gastric lipoma [1,2,12]. Therefore, if a large submucosal tumor is detected on an endoscopic or UGI examination, then a CT scan should be obtained for making the diagnosis and deciding on the method of therapy, like in our case.

Conservative treatment is preferred for solitary lipomas that are asymptomatic. However, further evaluation and surgical management should be planned when endoscopic examination shows an ulcer or when tumors contain non-fatty elements, are symptomatic or show infiltrative growth patterns [1,2,4,9,12,15,19]. The current patient presented with non-specific gastrointestinal symptoms and endoscopic examination ruled out the possibility of mucosal ulceration or bleeding. A decision was made to followed-up due to the morbidity of a total gastrectomy [1,2,6,20].

## 4 Conclusion

Here, we report a very rare case of gastric lipomaosis, medical and follow up management is appropriate when gastric lipomatosis is asymptomatic without mucosal ulceration and in asymptomatic patient, regardless of the amount of gastric lipomas, avoiding patient the morbidity of a total gastrectomy.

## Declaration of Competing Interest

Nothing to declare.

## Sources of funding

Nothing to declare.

## Ethical approval

The study is exempt from ethnical approval in our institution.

## Consent

Written informed consent was obtained from the patient for publication of this case report.

## Author contribution

Dr. Solano, Dr Herrera and Dr Cadena: Evaluation the case along with analysis.

Dr. Solano, Dr. Cabrera, Dr Isaac: Performed the endosocpic approach.

Dr. Pedraza: Assisted the endosocpic procedure.

## Registration of research studies

N/A.

## Guarantor

Mauricio Pedraza Ciro Mpedraza93@gmail.com.

## Provenance and peer review

Not commissioned, externally peer-reviewed.

## References

[1] Aoyama S., Ami K., Fukuda A., Imai K., Chong J., Ando M. (2017). Surg. Case Rep..

[2] Taylor A.J., Stewart E.T., Dodds W.J. (1990). Gastrointestinal lipomas: a radiologic and pathologic review. AJR Am. J. Roentgenol..

[3] Jeong I.H., Maeng Y.H. (2010). Gastric lipomatosis. J. Gastric Cancer.

[4] Thompson W.M. (2005). Imaging and findings of lipomas of the gastrointestinal tract. AJR Am. J. Roentgenol..

[5] Weinberg T., Feldman M. (1955). Lipomas of the gastrointestinal tract. Am. J. Clin. Pathol..

[6] Deeths T.M., Madden P.N., Dodds W.J. (1975). Multiple lipomas of the stomach and duodenum. Am. J. Dig. Dis..

[7] Ventura L., Leocata P., Guadagni S., Ventura T. (1997). Multiple gastric lipomas: report of an asymptomatic case found at autopsy. Pathol. Int..

[8] Kim H.S., Noh S.H., Kim C.K. (1989). Lipomas of gastrointestinal tract. J. Korean Surg. Soc..

[9] Ferrozzi F., Tognini G., Bova D., Pavone P. (2000). Lipomatous tumors of the stomach: CT findings and differential diagnosis. J. Comput. Assist. Tomogr..

[10] Suárez Moreno R.M., Hernández Ramírez D.A., Madrazo Navarro M., Salazar Lozano C.R., Martínez Gen R. (2010). Multiple intestinal lipomatosis. Case report. Cir. Cir..

[11] Peabody J.W., Zikind J. (1953). Lipomatosis of the stomach; a case report and a review of the literature. Ann. Surg..

[12] Ventura L., Leocata P., Guadagni S., Ventura T. (1997). Multiple gastric lipomas: report of an asymptomatic case found at autopsy. Pathol. Int..

[13] Skinner M.S., Broadaway R.K., Grossman P., Seckinger D. (1983). Multiple gastric lipomas. Dig. Dis. Sci..

[14] Devlies F., Hoe L.V., Leemans A., Ponette E., Paepe I.D. (1997). Gastroduodenal lipomatosis. Eur. Radiol..

[15] Cabaud P.G., Harris L.T. (1959). Lipomatosis of the ileocecal valve. Ann. Surg..

[16] Fawcett N.W., Bolton V.L., Geever E.F. (1949). Multiple lipomas of the stomach and duodenum. Ann. Surg..

[17] Weinberg T., Feldman M. (1955). Lipomas of the gastrointestinal tract. Am. J. Clin. Pathol..

[18] Thompson W.M., Kende A.I., Levy A.D. (2003). Imaging characteristics of gastric lipomas in 16 adult and pediatric patients. AJR Am. J. Roentgenol..

[19] Siegal A., Witz M. (1991). Gastrointestinal lipoma and malignancies. J. Surg. Oncol..

[20] The Information Committee of the Korean Gastric Cancer Association (2008). 2005–2006 nationwide gastric submucosal tumor report in Korea. J. Korean Gastr. Cancer Assoc..

[21] Agha R.A., Borrelli M.R., Farwana R., Koshy K., Fowler A., Orgill D.P., For the SCARE Group (2018). The SCARE 2018 statement: updating consensus Surgical Case Report (SCARE) guidelines. Int. J. Surg..

